# Endoscopic Diagnosis of Gastric Subepithelial Lesions < 20 mm: Current Strategies and Emerging Solutions

**DOI:** 10.1111/den.70079

**Published:** 2026-01-11

**Authors:** Yosuke Minoda, Shuzaburo Nagatomo, Haruei Ogino, Nao Fujimori, Eikichi Ihara

**Affiliations:** ^1^ Department of Medicine and Bioregulatory Science, Graduate School of Medical Sciences Kyushu University Fukuoka Japan; ^2^ Department of Endoscopic Diagnostics and Therapeutics Kyushu University Hospital Fukuoka Japan; ^3^ Department of Gastroenterology and Metabolism, Graduate School of Medical Sciences Kyushu University Fukuoka Japan

**Keywords:** endoscopic ultrasound‐guided tissue acquisition, endosonography, fine‐needle biopsy, gastrointestinal stromal tumor, subepithelial lesion

## Abstract

Gastric subepithelial lesions (SELs) < 20 mm are frequently identified during routine endoscopy and account for approximately 90% of all SELs. Although most are benign, a substantial proportion represents gastrointestinal stromal tumors (GISTs), which carry malignant potential even at this small size. Histological confirmation is critical for appropriate risk assessment and treatment planning. However, the diagnostic yield of endoscopic ultrasound‐guided tissue acquisition (EUS‐TA) is limited for SELs < 20 mm due to technical challenges such as lesion mobility and short needle stroke. Mucosal incision–assisted biopsy (MIAB), which enables direct visualization and targeted sampling, has emerged as a practical alternative. This narrative review summarizes current evidence on endoscopic diagnostic approaches for SELs < 20 mm, including both sampling methods (EUS‐TA, MIAB) and nonsampling techniques such as contrast‐enhanced EUS, elastography, and artificial intelligence (AI)‐assisted image analysis. Each modality has distinct advantages and limitations, and selection should be based on lesion characteristics, endoscopist experience, and resource availability. Nonsampling modalities offer complementary information and are expected to become increasingly relevant. A comprehensive understanding of available diagnostic techniques is essential to support accurate clinical decision‐making for SELs < 20 mm.

## 1 Introduction

Subepithelial lesions (SELs) of the stomach are detected in 0.5%–3.0% of routine endoscopic examinations [1, 2, 3, 4]. SELs < 20 mm account for approximately 90% of all SELs, although the exact proportion depends on the study population and diagnostic modality [4, 5, 6]. Most of these small SELs have been reported benign; nevertheless, approximately 25%–50% have been identified as gastrointestinal stromal tumors (GISTs) [7, 8, 9], which retain malignant potential even at this size [10, 11, 12].

Endoscopic ultrasound‐guided tissue acquisition (EUS‐TA) has been the gold standard for histological diagnosis. However, its diagnostic yield falls for SELs < 20 mm because accessing and sampling such small targets is technically challenging [7, 13, 14, 15, 16]. In real‐world practice, gastric SELs < 20 mm are typically followed with endoscopic surveillance, in accordance with recommendations in several guidelines [17, 18, 19]. Recent advancements in EUS‐FNB needle design, novel EUS imaging techniques—including contrast‐enhanced [20] and artificial intelligence (AI)‐assisted modalities—and the expanding feasibility of minimally invasive endoscopic resection have prompted renewed interest in the optimal management of gastric SELs < 20 mm. Although current guidelines acknowledge the need for individualized decision‐making, they offer limited specific recommendations for this size category, underscoring the importance of reassessing diagnostic and therapeutic strategies [17]. This review aims to comprehensively examine endoscopic diagnostic approaches for gastric SELs < 20 mm, delineating the advantages, limitations, and challenges associated with conventional techniques, while also summarizing the current development and clinical potential of emerging diagnostic modalities.

## 2 Methods

We conducted a narrative, semi‐systematic review of the English literature on gastric SELs < 20 mm. Two databases—PubMed and the Cochrane Library—were searched from January 2000 to April 2025. For each diagnostic domain, we applied a tailored Boolean string that combined free text and controlled vocabulary; the core concepts were (i) mucosal‐incision/unroofing/single‐incision needle‐knife (SINK) biopsy, (ii) fine‐needle aspiration (EUS‐FNA) or fine‐needle biopsy (EUS‐FNB) with size terms (“small,” “< 20 mm”), (iii) harmonic or contrast‐enhanced EUS (CE‐EUS), (iv) endoscopic elastography, including shear wave and strain elastography, (v) artificial intelligence/deep learning applied to EUS, (vi) needle‐based confocal laser endomicroscopy (cCLE), and (vii) conventional EUS morphology (full syntax provided in Table S1).

After automatic de‐duplication, YM screened titles and abstracts, retrieved full texts, and resolved disagreements by consensus. Reference lists of all eligible papers were hand‐searched. We included original studies and case series reporting diagnostic performance and/or safety. Case reports with fewer than 10 patients and nonpeer‐reviewed abstracts were excluded. For each study we extracted design, sample size, accuracy, sensitivity, specificity, and complications. Evidence quality was categorized as randomized controlled trial (RCT), prospective or retrospective studies. The review conforms to the SANRA checklist for narrative reviews and adheres to PRISMA‐ScR guidance where applicable.

### 2.1 Search Results

**FIGURE 1:**
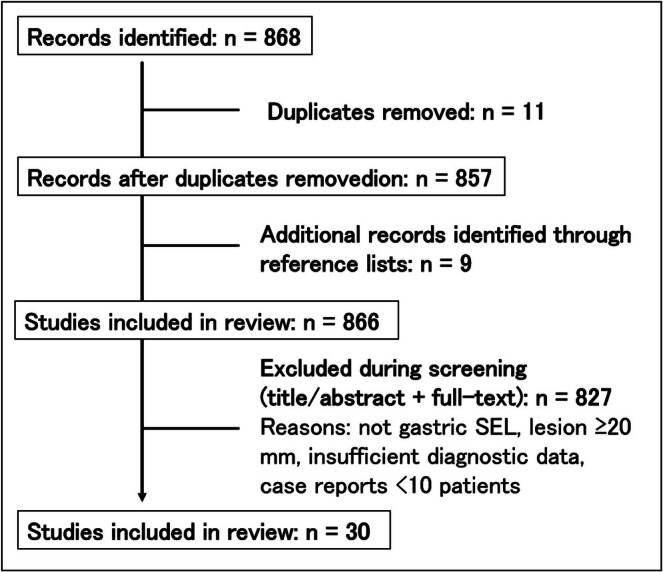
Flowchart to literature selection.

A total of 868 records were initially identified through electronic database searches. After the removal of duplicates, 857 records remained for title and abstract screening. In addition, nine relevant articles were identified through manual screening of reference lists. After full‐text assessment based on predefined inclusion and exclusion criteria, 30 studies met our criteria (conventional EUS 1, EUS‐TA 14, mucosal‐incision–assisted biopsy (MIAB) 10, CE‐EUS 2, AI‐assisted EUS (AI‐EUS) 3, nCLE 0) (Figure 1). The commonest exclusions were small case series (< 10 patients) and lack of diagnostic data. Nine additional papers were identified via reference‐list screening.

### 2.2 Overview of Guidelines Strategy for SELs < 20 mm

Current guidelines generally permit follow‐up for gastric SELs < 20 mm, endorsing a watch‐and‐wait strategy in the absence of high‐risk features. Tissue sampling is typically considered for lesions that exhibit concerning imaging findings or interval growth. When sampling is indicated, endoscopists can choose among several techniques such as EUS‐TA and MIAB.

### 2.3 Natural History of SELs < 20 mm

The natural history of gastric SELs < 20 mm is generally indolent. Several cohort studies have shown that more than 90% of these lesions remain stable during several years of follow‐up, and only approximately 5%–10% exhibit measurable growth [21, 22]. Growth has been reported to be uncommon in lesions < 10 mm, whereas the risk increases markedly in those ≥ 13.5 mm [23, 24]. Other reported risk factors include irregular or lobulated margins, mucosal ulceration, and cystic changes on EUS [25, 26]. Thus, most SELs < 20 mm can be safely managed by endoscopic surveillance. However, certain lesions may warrant histological diagnosis depending on their imaging features and clinical context.

### 2.4 Indications for Histological Diagnosis

The indication for histological diagnosis in gastric SELs < 20 mm requires careful consideration. Lesions such as cysts and lipomas exhibit characteristic EUS features. By contrast, when gastrointestinal mesenchymal tumors (GIMTs) including GISTs cannot be excluded, histological confirmation becomes important not only to establish a definitive diagnosis but also to guide treatment strategy. In this context, EUS morphology provides valuable initial stratification, while histology remains essential in lesions where the biological behavior cannot be confidently predicted from imaging alone.

### 2.5 Conventional EUS Morphology

**FIGURE 2:**
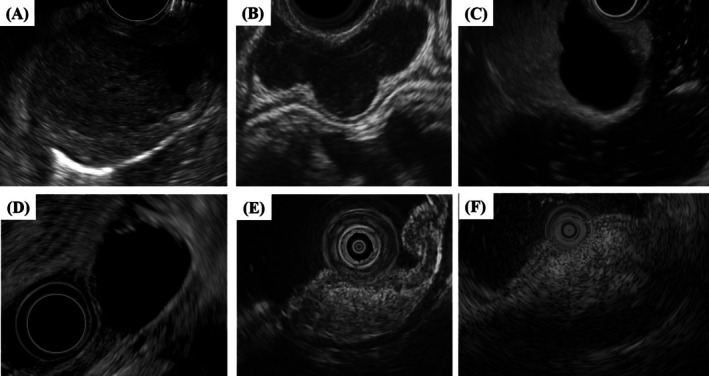
Representative EUS images of various SELs: (A) Gastrointestinal stromal tumor, (B) leiomyoma, (C) compression from liver cyst, (D) cyst, (E) lipoma, and (F) aberrant pancreas.

Classic features—layer of origin, echogenicity, border regularity, and intratumoral heterogeneity—remain the backbone of imaging‐based risk assessment [17, 27, 28, 29]. GIMTs, including GISTs and leiomyomas, typically originate from the muscularis propria. Hypoechoic lesions arising from the fourth hypoechoic layer with lobulated margins, cystic spaces, or ulceration raise particular concern for GIST or other malignant entities (Figure 2A), whereas leiomyomas often show more homogeneous echogenicity with elongated, well‐defined margins (Figure 2B). Nevertheless, even in expert hands the stand‐alone diagnostic accuracy of EUS morphology is only about 50%, and interobserver variability together with feature overlap between benign and malignant lesions further limits its reliability (Tables 1, 2, 3) [30]. Despite these limitations, EUS continues to play an indispensable role in determining whether to perform specimen sampling, and in confirming that a subepithelial‐appearing protrusion is not due to compression from adjacent extraluminal structures (Figure 2C). Importantly, certain lesions such as cysts, lipomas, and aberrant pancreas can be reliably identified based on their characteristic EUS features, which often provide sufficient diagnostic confidence to preclude the need for invasive tissue acquisition [31]. Typically, cysts appear as well‐demarcated anechoic structures (Figure 2D), and lipomas as homogeneously hyperechoic lesions (Figure 2E). Aberrant pancreas is also characterized by its origin in the submucosal layer and may occasionally exhibit focal thickening of the muscularis propria (Figure 2F).

**TABLE 1:** Summary of diagnostic yield of main endoscopic modalities for SEL < 20 mm.

Modality	Accuracy	Sensitivity/specificity	Advantages	Limitations	Clinical availability	Evidence volume
EUS alone	46%	31%/67%	Less‐invasive	Low accuracy	Universal	Limited
EUS‐TA	35%–84% (FNA) 83%–100% (FNB)	NR/83%–100%^a^	High specificity	Technical difficulty	Widely available	Extensive
MIAB	78%–100%	NR/100%^a^	High accuracy and specificity	Technically demanding	Available in some centers	Moderate
CE‐EUS	85%–93%	89%/83%	Simple	Unstandardized interpretation	Available in some centers	Limited
EUS‐SE	87%	89/83%	Less‐invasive	Unstandardized interpretation	Available in some centers	Limited
EUS‐SWE	80%	78/83%	Less‐invasive	Unstandardized interpretation	Available in some centers	Limited
AI‐EUS	86%–94%	86%–93%/62%–100%	Objective, rapid	Research use only	Research setting	Limited

^a^ Specificity values for EUS‐TA and MIAB were assessed in cases with final histological diagnosis based on resected specimens.

### 2.6 Endoscopic Sampling Approach

**FIGURE 3:**
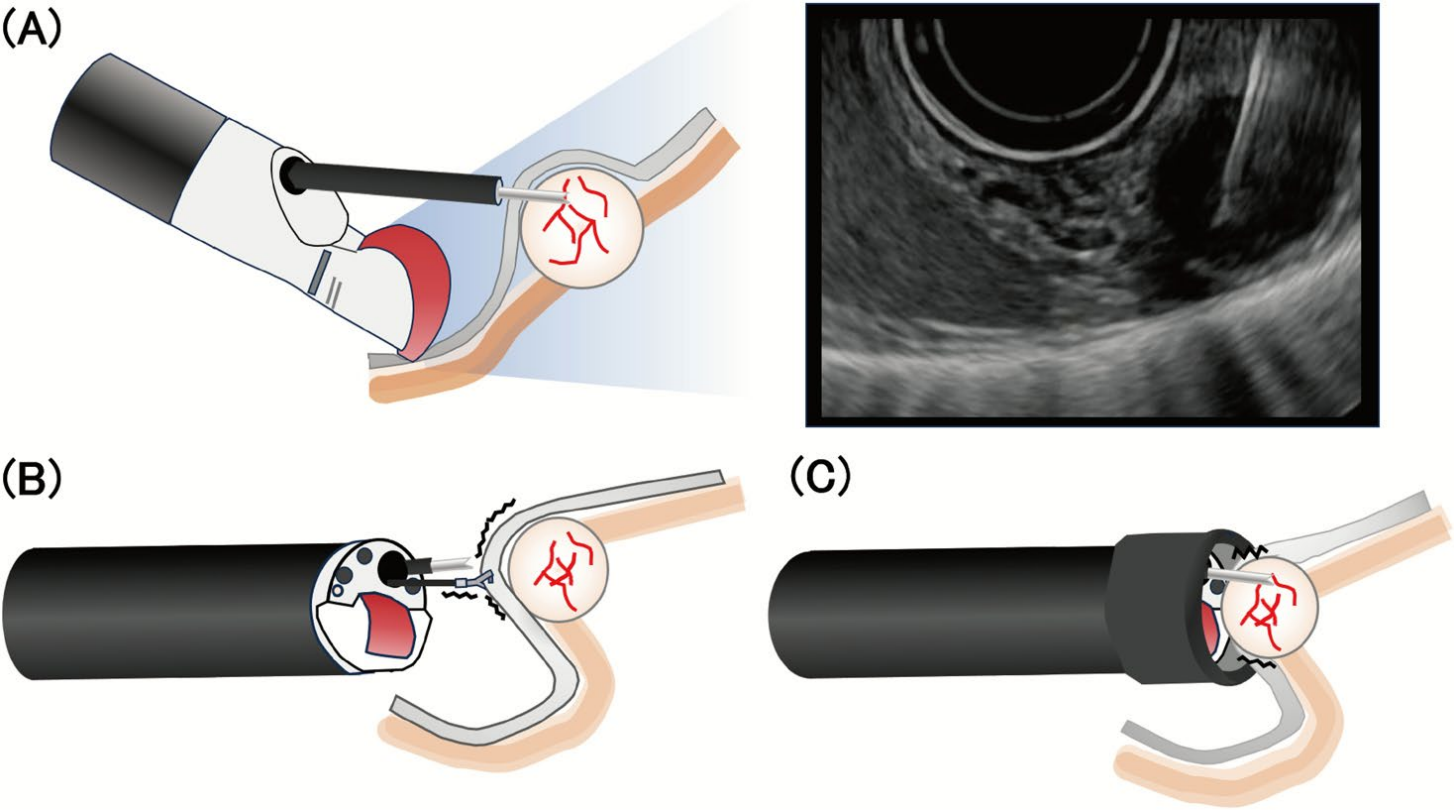
Schematic illustration of EUS‐TA. (A) Conventional EUS‐TA, (B) traction‐assisted EUS‐TA, and (C) cap assisted EUS‐TA.

EUS‐TA is widely regarded as the standard and safe method for histological diagnosis of gastric SELs < 20 mm [32]. MIAB, an approach adapted from endoscopic submucosal dissection, has recently gained attention as a practical alternative when en‐bloc core tissue is required [20]. The following subsections outline the key procedural features, strengths, and limitations of each technique (Figure 3).

### 2.7 Technics of EUS‐TA

#### 2.7.1 Overview of EUS‐TA

EUS‐TA is generally categorized into EUS‐FNB and EUS‐FNA. EUS‐FNB needles incorporate design modifications that improve tissue‐capture efficiency, and numerous studies in the pancreatobiliary field have shown remarkably high sampling yields. Although FNB needles retrieve larger histological cores than conventional FNA needles, their penetration ability is generally considered lower than that of FNA needles.

#### 2.7.2 Diagnostic Performance of EUS‐TA

Although the optimal device settings for EUS‐TA in SELs < 20 mm have not been clearly established, it is common practice to use a 22‐gauge needle with dry suction during the procedure (Table 2). Because SELs < 20 mm allow only a short needle track inside the lesion, the to‐and‐fro stroke needed to obtain an adequate core is necessarily short. Even after multiple passes, the needle often retrieves fragmented or insufficient material, with reported rates ranging from 35% to 84% across published series (Tables 1, 2, 4 and 5) [7, 8, 14, 15, 33, 34, 35, 36, 37, 38, 39, 40, 41, 42]. The short working distance also amplifies the impact of even slight targeting errors, further compromising specimen quality. While many studies report that EUS‐FNB achieves higher diagnostic accuracy than EUS‐FNA for SELs < 20 mm, some studies have demonstrated only modest or negligible differences; consequently, the evidence remains inconclusive (Tables 4 and 5) [15, 40, 42, 43, 44, 45].

**TABLE 2:** Procedure time and adverse event of modalities for SELs < 20 mm.

Modality	Estimated procedure time	Complications
EUS alone	5–10 min	0%
EUS‐TA	20 min	< 0.5%–1%; predominantly minor bleeding, very rare perforation
MIAB	30 min	3%–5%, controllable intra‐procedural bleeding, occasional delayed bleeding
CE‐EUS	10–15 min (including baseline EUS)	0%
EUS‐SE	10–15 min (including baseline EUS)	0%
EUS‐SWE	10–15 min (including baseline EUS)	0%
AI‐EUS	5–10 min (practically real‐time)	0% (software‐based)

Abbreviations: AI‐EUS, artificial intelligence‐assisted EUS; CE‐EUS, contrast‐enhanced EUS; EUS‐SE, strain elastography; EUS‐SWE, shear‐wave elastography; EUS‐TA, endoscopic ultrasound‐tissue acquisition; MIAB, mucosal incision‐assisted biopsy; NR, not reported.

**TABLE 3:** Diagnostic yield of EUS alone for SEL < 20 mm.

Author (year)	Country	Cases	Accuracy (%)	Sensitivity (%)	Specificity (%)	Complication (%)	Evidence level
Karaca (2010) [30]	USA	22	46	31	67	0	Retrospective

**TABLE 4:** Diagnostic yield of EUS‐TA for SEL < 20 mm.

Author (year)	Country	Cases	Needle type	Accuracy	Specificity^a^	Complication	Evidence level
Akahoshi (2014) [7]	Japan	21	FNA	71%	98%	0%	Retrospective
Sekine (2015) [39]	Japan	19	FNA	81%	100%	0%	Retrospective
Kobara (2017) [33]	Japan	17	FNA	35%	NR	0%	Prospective
Attila (2018) [38]	Turkey	10	FNA	50%	83%	4.5%	Retrospective
Osoegawa (2019) [35]	Japan	13	FNA	54%	100%	0%	RCT
Kobayashi (2024) [34]	Japan	104	FNA	84%	NR	0%	Retrospective
Iwai (2018) [14]	Japan	6	Both	83% (FNA) 83% (FNB)	100%	0%	RCT
Fujita (2018) [36]	Japan	15	Both	60% (FNA) 100% (FNB)	NR	0%	Retrospective
Trindada (2019) [37]	USA	23	Both	39% (FNA) 82% (FNB)	NR	0	Prospective
Minoda (2020) [40]	Japan	56	Both	68% (FNA) 78% (FNB)	100%	0%	Retrospective
Sekine (2021) [15]	Japan	24	Both	73% (FNA) 87% (FNB)	NR	0%	Retrospective
Inoue (2019) [42]	Japan	30	Both	67%	NR	4%	Retrospective
Minoda (2025) [8]	Japan	30	FNB	67%	100%	0%	RCT

Abbreviations: AUC, area under the curve; CE‐EUS, contrast‐enhanced endoscopic ultrasound; EUS‐TA, endoscopic ultrasound‐tissue acquisition; MIAB, mucosal incision‐assisted biopsy; NR, not reported.

^a^ Specificity values for EUS‐TA and MIAB were assessed in cases with final histological diagnosis based on resected specimens.

**TABLE 5:** Detail of EUS‐TA of Table 4.

Author (year)	Needle type	Needle brand(s)	Gauge	Suction method
Akahoshi (2014) [7]	FNA	NA‐11J‐KB	22	Dry suction (20 mL)
Sekine (2015) [39]	FNA	NA‐10J‐1, NA‐10J‐B, NA‐200H‐8022, Expect, Echo tip	22/25	NR
Kobara (2017) [33]	FNA	Expect	19/22/25	Dry suction (10 mL)
Attila (2018) [38]	FNA	NR	22	NR
Osoegawa (2019) [35]	FNA	Expect, Sono Tip, EZ Shot 3, EchoTip	20/22/25	Dry suction
Kobayashi (2024) [34]	FNA	Acquire, Expect, EZ Shot 3 Plus	19/22	NR
Iwai (2018) [14]	Both	EchoTip Ultra (FNA) EchoTip ProCore (FNB)	19/22	Dry suction (10 mL)
Fujita (2018) [36]	Both	Expect (FNA) Acquire (FNB)	22	Dry suction (20 mL)
Trindada (2019) [37]	Both	NR (FNA) Sharkcore (FNB)	19/22/25	Dry suction (10 mL)
Minoda (2020) [40]	Both	Expect, Sono Tip, EZ Shot 3 (FNA) Acquire, Echo Tip Procore (FNB)	19/20/22/25	Dry suction (20 mL)
Sekine (2021) [15]	Both	Expect, EZshot3, Sono Tip (FNA) Acquire, Echo Tip Procore (FNB)	19/20/22/25	Dry suction (20 mL)
Inoue (2019) [42]	Both	Expect, Echo Tip Ultra, EZ shot (FNA) Acquire, Echo Tip ProCore (FNB)	19/22/25	Dry suction (10 or 20 mL)
Minoda (2025) [8]	FNB	Acquire	22	Dry suction (20 mL)

Abbreviation: NR, not reported.

**TABLE 6:** Diagnostic yield of mucosal incision–assisted biopsy for SEL < 20 mm.

Author (year)	Country	Cases	Accuracy (%)	Specificity^a^	Complication	Evidence level
Lee (2010) [46]	Korea	16	94	NR	0%	Prospective
Ihara (2013) [47]	Japan	27	85	100%	0%	Retrospective
Binmoeller (2014) [48]	USA	23	100	NR	0%	Retrospective
Shimamura (2017) [49]	Canada	18	89	NR	8%; minor bleeding	Retrospective
Kobara (2017) [33]	Japan	29	100	100%	0%	Prospective
Park (2019) [41]	Korea	12	80	NR	0%	Prospective
Osoegawa (2019) [35]	Japan	23	91	100%	0%	RCT
Minoda (2020) [40]	Japan	45	93	100%	0%	Retrospective
Nakano (2020) [50]	Japan	45	78	100%	2%; perforation and bleeding	Retrospective
Wong (2022) [51]	Taiwan	50	94	NR	2%	Prospective

Abbreviation: NR, not reported.

^a^ Specificity values were assessed in cases with final histological diagnosis based on resected specimens.

**TABLE 7:** Comparison of diagnostic yield between EUS‐TA and MIAB.

Author (year)	Country	Case (< 20 mm)	Lesion size (< 20 mm data availability)	Accuracy (MIAB/EUS‐TA)	Procedure time (MIAB/EUS‐TA)	Complications	Evidence level
Ikehara (2015) [52]	Japan	7	Yes (stratified)	MIAB; 75% EUS‐TA; 58.3%	NR (no sub‐group data)	0%	RCT
Kobara (2017) [33]	Japan	14	Mixed (no sub‐group)	NR (No subgroup data)	NR (no sub‐group data)	0%	Prospective
Osoegawa (2019) [35]	Japan	24	Mixed (no sub‐group)	MIAB; 90.9% EUS‐TA; 53.9%	NR (no sub‐group data)	0%	RCT
Park (2019) [41]	Korea	30	Mixed (no sub‐group)	MIAB; 64.3% EUS‐TA; 78.6%	NR (no sub‐group data)	NR (no sub‐group data)	Prospective
Minoda (2020) [40]	Japan	101	Yes (stratified)	MIAB; 93.3% EUS‐TA; 71.4%	MIAB; 31 min EUS‐TA; 20 min	0%	Retrospective

Abbreviations: EUS‐TA, endoscopic ultrasound–guided tissue acquisition; FNA, fine‐needle aspiration; FNB, fine‐needle biopsy; MIAB, mucosal incision–assisted biopsy; NR, not reported.

**TABLE 8:** Diagnostic yield of contrast‐enhanced EUS for SEL < 20 mm.

Author (year)	Country	Cases	Accuracy (%)	Sensitivity (%)	Specificity (%)	Complications	Evidence level
Kamata (2017) [53]	Japan	20	85	85	100	NR	Retrospective
Yamazaki (2024) [54]	Japan	15	93	89	88	NR	Retrospective

Abbreviations: EUS, endoscopic ultrasound; NR, not reported; SEL, subepithelial lesion.

**TABLE 9:** Diagnostic yield of AI‐assisted EUS for SEL < 20 mm.

Author (year)	Country	Cases	Target modality	Accuracy	Sensitivity	Specificity	Evidence level
Minoda (2020) [55]	Japan	30	EUS images	86%	86%	63%	Retrospective
Tanaka (2022) [56]	Japan	17	CE‐EUS images	94%	93%	100%	Retrospective
Duan (2025) [57]	China	Internal validation	EUS images	Only AUC is shown	NR	NR	Retrospective

Abbreviations: AI, artificial intelligence; AUC, area under the curve; CE‐EUS, contrast‐enhanced endoscopic ultrasound; NR, not reported; SEL, subepithelial lesion.

#### 2.7.3 Technical Considerations and Tips

Small SELs frequently shift with respiration or with slight endoscope torque, a phenomenon often called “target drift,” which diminishes effective penetration and requires the endoscopist to realign repeatedly [58, 59]. Clinical experience suggests that the EUS scope cannot always be kept in firm contact with these small, mobile lesions, making a stable needle trajectory difficult to maintain. As a result, multiple repositioning attempts are often anticipated, each potentially lengthening the procedure and increasing the likelihood of an inadequate specimen.

#### 2.7.4 Current Limitations and Reported Refinements to Overcome “Target Drift”

##### 2.7.4.1 Traction‐Assisted Fixation

A clip‐with‐line is used to pull the lesion firmly against the gastric wall over the SEL, reducing target drift and lengthening the usable needle stroke [59] (Figure 3B). Although diagnostic yields were reported roughly 85%–90%, the technique demands extra devices [8].

##### 2.7.4.2 Cap‐Assisted Fixation

A transparent cap or suction hood encloses the lesion and stabilizes the needle tip (Figure 3C). This approach achieves diagnostic yields comparable to traction methods; however, the requirement for a forward‐viewing echoendoscope (FV‐EUS) limits its widespread adoption [33].

Both fixation techniques have demonstrated improvements in diagnostic yield; however, their reliance on specialized endoscopes or accessories and the associated procedural complexity remain limiting factors. Furthermore, robust comparative data evaluating their relative efficacy are lacking. Ongoing innovation focused on the development of simplified, broadly accessible stabilization devices or high‐penetration needles is therefore critical to facilitating effective tissue acquisition in SELs < 20 mm.

### 2.8 Technics of MIAB

#### 2.8.1 Overview of MIAB

**FIGURE 4:**
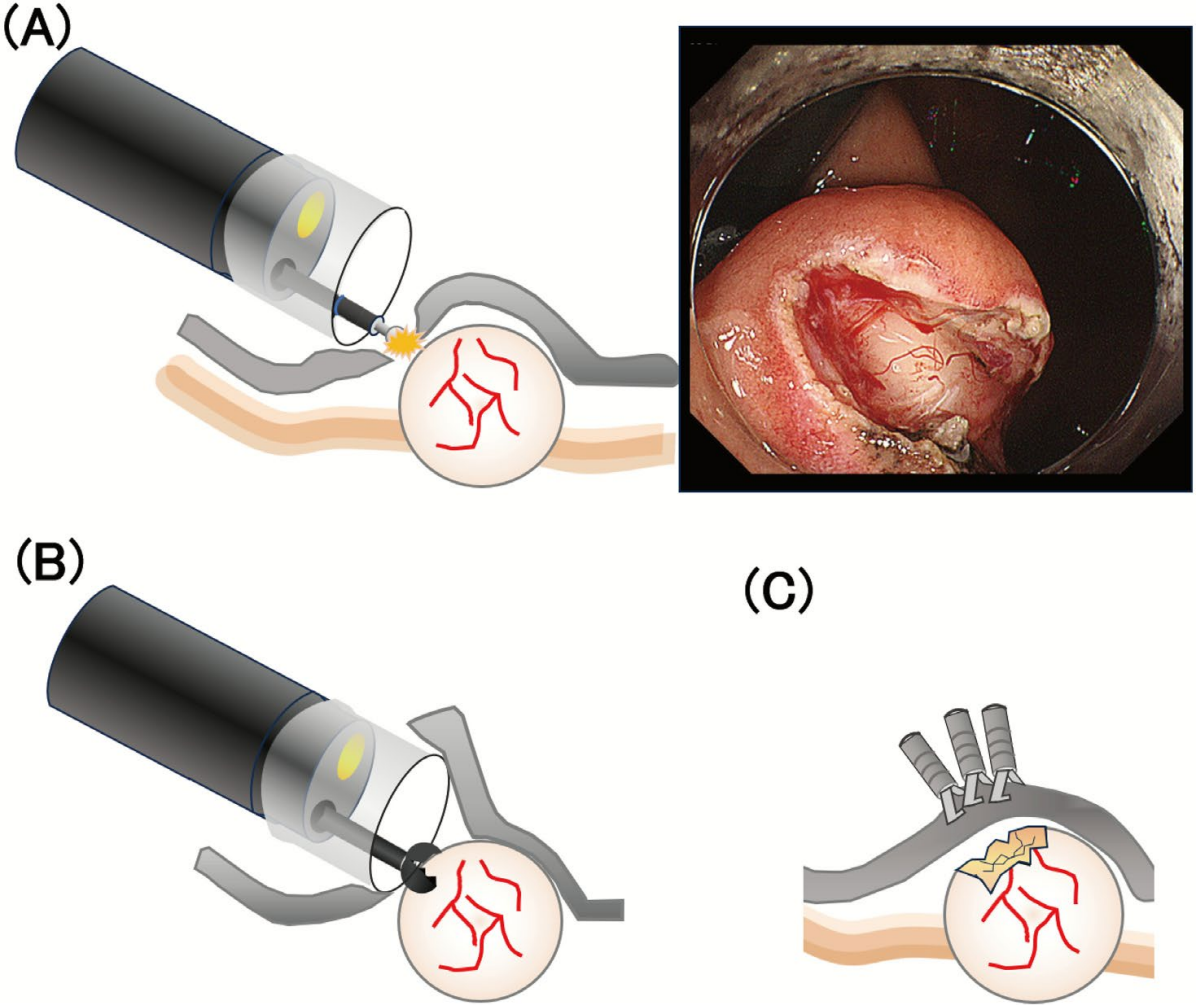
Schematic illustration of mucosal incision‐assisted biopsy. (A) Mucosal incision for the mucosa over SELs, (B) biopsy for the lesions, and (C) closure of incised mucosa.

When EUS‐FNB fails or is infeasible, MIAB offers a direct‐vision approach to tissue acquisition (Figure 4). A similar technique, described in the literature as “SINK biopsy,” follows the same principle of exposing the lesion surface to allow forceps to obtain a core specimen [46, 47, 52, 60, 61]. While some studies distinguish between MIAB and SINK biopsy, others use the terms interchangeably or consider them conceptually related under the broader category of mucosal incision–assisted biopsy techniques. In this review, we use the term “MIAB” in a broad sense to encompass direct‐vision forceps biopsy techniques involving mucosal incision, including what has been described in the literature as “SINK biopsy.”

#### 2.8.2 Diagnostic Performance

Because the endoscopist can visualize the target directly, diagnostic success is independent of tumor size [62]. Multicenter series consistently report diagnostic accuracy of 78%–100%, and sufficient material is usually obtained for both histology and immunohistochemistry, even in small GISTs (Tables 1, 2 and 6) [33, 35, 40, 41, 46, 47, 48, 49, 50, 51].

#### 2.8.3 Technical Considerations and Tips

Successful MIAB requires precise and careful execution of several key steps. The mucosal incision must be placed directly over the lesion; incisions that are misaligned or obliquely oriented can result in incomplete lesion exposure or inadvertent perforation [63].

#### 2.8.4 Current Limitations and Future Refinements

MIAB is technically demanding and is primarily performed at centers with advanced expertise in therapeutic endoscopy. When a large mucosal incision is required, closing the resulting defect can be challenging; however, the recent availability of wide‐opening clips and the combination of endoloops with standard clips now permits safe and reliable defect closure [64, 65, 66, 67, 68, 69, 70]. As equipment and training continue to improve, MIAB is likely to mature into a size‐independent, direct histologic strategy for gastric SELs < 20 mm.

### 2.9 Selection of MIAB Versus EUS‐TA Based on Lesion Size

Although only a limited number of studies directly compare EUS‐TA with MIAB, the available evidence suggests a diagnostic crossover point at approximately 12–15 mm. Lesions smaller than this threshold tend to be more accurately diagnosed by MIAB, with reported diagnostic yields of 75%–93% in comparative studies, whereas EUS‐TA achieves yields of 54%–79%. Procedure time is typically longer with MIAB (approximately 30 min) compared to EUS‐TA (approximately 20 min) [35] (Table 7). However, the optimal strategy for lesions in which the initial technique (EUS‐TA or MIAB) has yet to be elucidated. Based on currently published data, the decision algorithm outlined below appears reasonable, but it should be considered provisional pending further prospective validation. Notably, the choice of either approach does not appear to significantly affect prognosis following surgical treatment [71].

### 2.10 Stepwise Redeployment of EUS‐TA and MIAB After Failure of an Initial Diagnostic Attempt

When the first tissue‐acquisition procedure fails, clinicians must decide the next approach: EUS‐TA, MIAB, or follow‐up. The following sections summarize the data underpinning each tactic and offer practical guidance on integrating EUS‐TA and MIAB in a sequential, patient‐tailored algorithm.

#### 2.10.1 EUS‐TA → EUS‐TA

Antonini et al. demonstrated that switching to a 25G core biopsy needle after initial FNB failure led to diagnostic success in 56% of patients [72]. While the literature specific to gastric SELs is limited, data from pancreatic lesion studies suggest that repeating EUS‐TA yields a diagnostic result in approximately 60%–80% of previously nondiagnostic cases [73, 74]. These reports support the idea that a second EUS‐TA, especially with adjusted technique (e.g., needle type, suction, number of passes), can salvage diagnostic accuracy.

#### 2.10.2 EUS‐TA → MIAB

Some studies support MIAB as a highly effective salvage technique following nondiagnostic EUS‐FNA/B. In a randomized crossover trial, Osoegawa et al. found that after initial EUS‐FNA failure, MIAB achieved a 71.4% diagnostic yield [35]. These findings support a tiered diagnostic approach starting with EUS‐FNB and escalating to MIAB when necessary.

#### 2.10.3 MIAB → EUS‐TA

Although technically more challenging, switching from MIAB to EUS‐TA is also feasible. In the same Osoegawa study, diagnostic yield was 50% in this strategy [35]. While this was lower than the MIAB rescue rate, it demonstrates that EUS‐TA remains an option even after prior MIAB, particularly if the lesion is not well‐exposed endoscopically.

#### 2.10.4 MIAB → MIAB

A second MIAB after an initial nondiagnostic attempt is conceptually feasible, yet its practicality and diagnostic value remain unsubstantiated. The first mucosal incision can induce fibrosis or alter the lesion's orientation, making re‐entry technically challenging and potentially hazardous. To date, no prospective or retrospective studies have reported the diagnostic yield or safety profile of repeat MIAB, so any recommendation is necessarily speculative.

**FIGURE 5:**
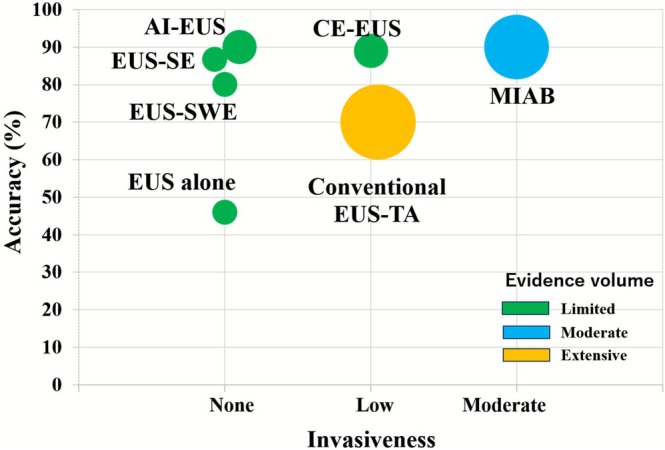
Evidence map of diagnostic modalities for gastric SELs < 20 mm based on accuracy, invasiveness, and evidence volume. Data summarized from Tables 1 and 2.

From a comparative standpoint, these approaches offer distinct diagnostic strengths depending on clinical context and lesion characteristics. Figure 5 illustrates these differences by comparing diagnostic accuracy, procedural invasiveness, and the level of supporting evidence across available modalities.

### 2.11 Endoscopic Nonsampling Approaches

Although morphologic assessment based on EUS images and other imaging modalities is an indispensable component of routine management, no nonsampling technique has yet demonstrated sufficient and reproducible accuracy to replace tissue acquisition as the definitive diagnostic standard. Nevertheless, several imaging‐based or physiologically informative techniques—such as those assessing vascularity, stiffness, or tissue perfusion—can refine risk stratification, guide the need for biopsy, and occasionally suggest a specific histology. Below we summarize the principal nonsampling methods to evaluate SELs.

### 2.12 Contrast Enhanced‐EUS/EUS Strain Elastography/EUS Share Wave Elastography

CE‐EUS evaluates lesion vascularity, with GISTs typically demonstrating hyperenhancement [75, 76]. Studies focusing on small SELs have reported diagnostic accuracy between 85% and 93% for GIST, though subjectivity and variability in interpretation remain challenges (Tables 1, 2 and 8) [53, 54]. Quantitative perfusion analysis is under development but not yet routine. Importantly, CE‐EUS does not require tissue puncture, making it well suited for initial risk stratification of small lesions.

Other EUS‐based evaluation methods, such as EUS strain elastography, EUS shear wave elastography, and DFI (detective flow imaging)‐EUS, have also been reported [77]. Although studies focusing specifically on SEL < 20 mm are limited, a report by Yamazaki et al. indicated that the accuracy of EUS strain elastography is approximately 87%, while the sensitivity of EUS‐SWE is around 80%. These modalities have the potential for combined use with CE‐EUS (Tables 1 and 2) [78].

### 2.13 AI‐Assisted Analysis of EUS Image

Recent studies have demonstrated that AI‐assisted analysis of EUS images can achieve diagnostic accuracy comparable to or even exceeding that of expert endosonographers, especially in distinguishing GISTs from non‐GIST [78, 79, 80, 81]. In a retrospective analysis, AI models achieved a sensitivity and specificity of approximately 90% and 80%, respectively, even for SELs < 20 mm (Tables 1, 2 and 9). These tools may offer the advantage of noninvasiveness, speed, and objectivity in the future [55, 56, 57, 82].

### 2.14 Others

#### 2.14.1 Needle‐Based Confocal Laser Endomicroscopy

nCLE provides real‐time optical biopsy by advancing a confocal mini‐probe through a 19‐gauge EUS needle [83]. Some studies have reported diagnostic accuracies of up to 88% for distinguishing GISTs from leiomyomas, particularly when lesions ≥ 20 mm are included [84, 85]. Nonetheless, the use of the technique is still limited to specialized institutions because a large‐bore needle and expert image interpretation are required. Puncturing SELs < 20 mm with a 19‐gauge needle could be particularly challenging, and dedicated prospective data on this size category are therefore still needed.

#### 2.14.2 Impedance‐Based Cellularity Assessment

Impedance measurement using high‐frequency alternating current is an experimental adjunct that estimates tissue cellularity by recording the electrical resistance encountered through a biopsy needle. To date, only one small pilot study has paired the technique with EUS‐FNB, yielding an overall sensitivity of 88.9% and specificity of 100% for distinguishing GISTs from other lesions [86]. Although these figures are favorable, size‐specific data were not reported, and the limited sample precludes firm conclusions. Larger, independent cohorts—especially with analyses focused on SELs < 20 mm—are still needed to establish clinical value.

#### 2.14.3 Clinical Implications and Future Directions

Advances in sampling techniques continue to shape the diagnostic landscape of gastric SELs. Next‐generation Franseen and fork‐tip FNB needles, refined traction or cap‐assisted stabilization methods, and streamlined MIAB protocols aim to reduce procedure time and complication rates while maintaining or improving diagnostic yield.

In parallel, nonsampling technologies such as CE‐EUS, nCLE, and impedance‐based cellularity assessment provide real‐time vascular, optical, or biophysical data. These modalities may complement histology when tissue is limited and can be deployed synergistically with EUS‐TA. Early studies suggest that such combinations may improve overall diagnostic accuracy and reduce nondiagnostic results.

AI systems for gastric SELs remain investigational; however, the broader field of medical AI or computer‐assisted systems is advancing rapidly [87]. Despite this progress, published data remain inconsistent: some studies report reduced accuracy in SELs < 20 mm, while others show stable performance. The reasons for this disparity—whether related to differences in training data sets, imaging protocols, or reference standards—are not yet clear. Should AI ultimately deliver reproducibly high accuracy for SELs < 20 mm, it may take on much of the diagnostic workload currently handled by EUS‐TA. Nonetheless, given the exceptionally high specificity of EUS‐TA, its confirmatory role will almost certainly remain essential.

Future research should prioritize standardized multicenter trials and consider the potential integration of AI with advanced imaging and physiologic or tissue‐characterization techniques, along with rigorous cost‐effectiveness analyses. Importantly, these innovations must be integrated into structured, patient‐centered diagnostic workflows—not simply substituted piecemeal—to enable high‐value, efficient clinical care.

## 3 Conclusion

The diagnostic challenge posed by gastric SELs < 20 mm remains a clinical challenge. Conventional EUS morphology provides important clues but lacks sufficient accuracy, while EUS‐guided tissue acquisition and MIAB achieve higher diagnostic yields. Emerging modalities, including CE‐EUS, elastography, and AI‐based approaches, hold potential but require further validation. From a clinical perspective, tissue acquisition has been the gold standard when histology is expected to guide management. Future studies should focus on refining minimally invasive techniques and validating AI‐driven strategies, with the ultimate goal of establishing an evidence‐based diagnostic algorithm tailored for small SELs.

## Author Contributions

Y.M. conceived and designed the review, conducted the literature search and analysis, and drafted the manuscript. All co‐authors reviewed the manuscript critically and provided intellectual input. All authors approved the final version of the manuscript.

## Funding

This work was partially supported by the National Cancer Center Research and Development Fund (grant no. 2023‐A‐15) and JSPS KAKENHI (grant no. 23K15044).

## Conflicts of Interest

Y.M. holds an R&D consulting contract with Olympus and MC medical. H.O. is part of an endowed course supported by several companies, including Ono Pharmaceutical, Miyarisan Pharmaceutical, Sanwa Kagaku Kenkyusho, Otsuka Pharmaceutical, Fujifilm Medica, Terumo Corporation, FANCL Corporation, Ohga Pharmacy, and Abbott Japan. E.I. has received lecture fees from Takeda Pharmaceutical, Viatris, and EA Pharma. Additionally, E.I. was part of an endowed course supported by these companies until March 2023. N.F. has received lecture fees from Boston Scientific. Y.O. has engaged in collaborative research with Fujifilm Medica and FANCL Corporation. The other authors declare no conflicts of interest for this article.

## Supporting information


**Table S1:** Search Terms for each endoscopic modality.
