# Supplementary material for: Endoscopic Diagnosis of Gastric Subepithelial Lesions < 20 mm: Current Strategies and Emerging Solutions
Source: Dig Endosc. 2026 Jan 11;38(1):e70079. doi: 10.1111/den.70079 (PMC12793713; doi:10.1111/den.70079)
Supplement: Supplementary file 1 — Table S1: Search Terms for each endoscopic modality. [file DEN-38-0-s001.docx]

Supplementary Table 1. Search Terms for each endoscopic modality

| Items | Search Terms |
| --- | --- |
| Conventional EUS | ("conventional EUS" OR "EUS morphology" OR "endosonographic features") AND (gastric OR stomach) AND (subepithelial OR SET OR SMT) |
| Contrast-enhanced / Harmonic EUS | ("contrast-enhanced" OR "harmonic") AND (EUS OR "endoscopic ultrasound") AND (gastric OR stomach) AND (subepithelial OR SET OR SMT) |
| Endoscopic elastography | "endoscopic elastography" or "Endoscopic shear wave elastogrpahy" or "Endoscopic strain elastography") AND (gastric OR stomach) AND (subepithelial OR SET OR SMT) |
| AI- EUS | (AI OR "artificial intelligence" OR "deep learning" OR CNN OR CADx OR CAD) AND (EUS OR "endoscopic ultrasound") AND (gastric OR stomach) AND (subepithelial OR SET OR SMT) |
| Confocal laser endomicroscopy | (nCLE OR "confocal laser endomicroscopy" OR endomicroscopy) AND (EUS OR "endoscopic ultrasound") AND (gastric OR stomach) AND (subepithelial OR SET OR SMT) |
| EUS-TA | (EUS-FNA OR EUS-FNB OR "fine-needle aspiration" OR "fine-needle biopsy") AND (gastric OR stomach) AND ("< 20 mm" OR small OR diminutive) AND (subepithelial OR SET OR SMT) |
| MIAB | ("mucosal-incision" OR "unroofing" OR "single-incision needle-knife" OR "SINK biopsy") AND (gastric OR stomach) AND (subepithelial OR SET OR SMT) |
